# Research Progress on Hydrogel–Elastomer Adhesion

**DOI:** 10.3390/ma15072548

**Published:** 2022-03-30

**Authors:** Lirong Meng, Jiang He, Caofeng Pan

**Affiliations:** 1 Center on Nanoenergy Research, School of Physical Science and Technology, Guangxi University, Nanning 530004, China; menglirong@foxmail.com (L.M.); cfpan@binn.cas.cn (C.P.); 2 CAS Center for Excellence in Nanoscience, Beijing Key Laboratory of Micro-Nano Energy and Sensor, Beijing Institute of Nanoenergy and Nanosystems, Chinese Academy of Sciences, Beijing 100083, China; 3 College of Physics and Optoelectronic Engineering, Shenzhen University, Shenzhen 518060, China; 4 School of Nanoscience and Technology, University of Chinese Academy of Sciences, Beijing 100049, China

**Keywords:** hydrogel–elastomer adhesion, adhesion mechanism, adhesion method, applications in bioelectronics

## Abstract

Hydrophilic hydrogels exhibit good mechanical properties and biocompatibility, whereas hydrophobic elastomers show excellent stability, mechanical firmness, and waterproofing in various environments. Hydrogel–elastomer hybrid material devices show varied application prospects in the field of bioelectronics. In this paper, the research progress in hydrogel–elastomer adhesion in recent years, including the hydrogel–elastomer adhesion mechanism, adhesion method, and applications in the bioelectronics field, is reviewed. Finally, the research status of adhesion between hydrogels and elastomers is presented.

## 1. Introduction

Hydrogels with varying structures and chemical compounds have different characteristics, including excellent transparency [1], high water content [2], excellent electrical conductivity [3], biocompatibility [4], biodegradability [5], etc. The unique characteristics of elastomers include stability in a variety of environments, mechanical solidity [6], and water resistance [7]. There is a natural need to integrate elastomers into hybrid structures that can potentially change their existing applications and achieve new functions because the advantages of elastomers and hydrogels are complementary. Based on this, the developed hydrogel–elastomer hybrid material-based device has a wide range of applications in the field of bioelectronics, including artificial muscle [8,9,10,11,12], artificial skin [13,14,15,16], and synthetic axons [17,18,19,20]. The poor adhesion between hydrophilic hydrogels (such as alginate/Ca^2+^, [21] κ-carrageen (CA), [22], and gelatin [23,24]) and hydrophobic elastomers is the biggest problem in developing hydrogel–elastomer hybrid-material-based equipment. In recent years, a large amount of progress has been made in achieving hydrogel–elastomer adhesion. Scholars have utilized various secondary non-covalent forces, including hydrogen bonding [25,26], hydrophilic and hydrophobic interactions [27], Van der Waals forces [28], and electrostatic interactions [29], to attain reversible and powerful adhesion between hydrogels and elastomer substrates. Adhesion has been interpreted via different developed mechanisms. For instance, given their complex-forming ability, hydrogels containing rich organic ligand groups are extremely adhesive to elastomers. Due to their powerful binding capacity and reversibility, hydrogen bonds are ideal non-covalent mechanisms for constructing multifunctional materials or for plasma or ozone treatment for hydrophobic elastomer surface modification so that the elastomer surface has a strong hydrophilic and hydrophobic effect, improving adhesion between the two. Compared to the breaking energy of hydrogels (high strength) and elastomers, the adhesion energy based on bonds (non-covalent) and substrates is usually considerably lower [30,31,32]. This confines their application in the field of bioelectronics [33,34,35]. Hence, the combination of non-covalent interaction and covalent interaction was proposed. Covalent bonding includes a silane bond [36], carbon–carbon bond [37], hydrazone bond [38], etc., which greatly improves the adhesion between hydrogels and elastomers. However, enhancing adhesion through surface modification [38] with a silane coupling agent is usually irreversible and has certain requirements on the functional groups of elastomers and hydrogels. Therefore, many recent studies have proposed a topological adhesion mechanism to improve adhesion. The topological bonding [39,40,41] mechanism does not require special functional groups between hydrogels and elastomers but makes the adhesion between hydrogels and elastomers reversible and is a widely studied adhesion mechanism. The synergistic effect of non-covalent mechanisms, covalent bonds, and topological bonding is usually adopted, which greatly enhances the adhesion energy and reduces many limitations [42,43,44] to improve hydrogel–elastomer adhesion as much as possible. Simultaneously, the application of hydrogel–elastomer hybrid material in artificial muscle, artificial skin, and the artificial axon is further expanded.

**Figure 1:**
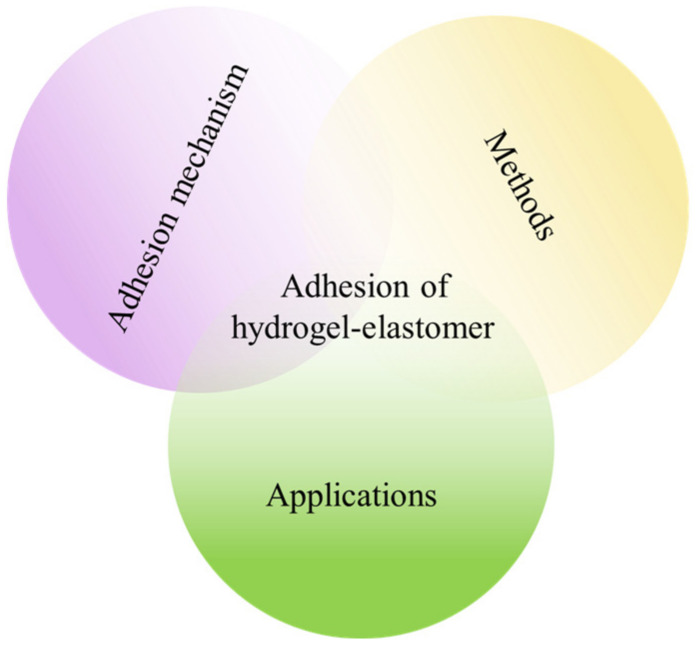
Schematic of the content of this review of hydrogel–elastomer adhesion.

In this paper, we review the research progress of hydrogel–elastomer adhesion and its application in bioelectronics (Figure 1). The second section introduces the adhesion mechanism of a representative hydrogel–elastomer. Section 3 highlights the regularly used adhesion methods under the three current adhesion mechanisms, including oxygen plasma or ozone treatment, catechol chemical treatment, surface modification, and topological bonding. The fourth section introduces the application of hydrogel–elastomer hybrid materials in bioelectronics, including but not limited to artificial muscle, artificial skin, and the artificial axon. Finally, the development prospect of the research on the adhesion between hydrogels and elastomers and its application in bioelectronics is presented. We hope to provide new insight into the challenges of hydrogel–elastomer bonding that exist in bioelectronics.

## 2. Hydrogel–Elastomer Adhesion Mechanism

Hydrogel–elastomer adhesion is mainly derived from non-covalent interaction, covalent interaction, and topological adhesion. Some non-covalent interactions are formed at the hydrogel–elastomer interface in the form of electrostatic interactions, Van der Waals forces, hydrophilic–hydrophobic interactions, hydrogen bonding, etc. This adhesion is weak but allows for repeated adhesion and stripping between hydrogels and elastomers, achieving reversibility of the adsorption process. Additionally, the covalent bonds created between hydrogels and elastomers (more commonly silane bonds) are usually strong but irreversible due to the limitations of the need to modify the functional groups of the hydrogels and elastomers. Finally, topological adhesion is based on the in situ formation of a spliced polymer network that is intertwined topologically with the polymer network of two pre-prepared materials, resulting in strong adhesion. This section emphasizes some of the main adhesion mechanisms at the interface between hydrogels and elastomers. The bonding between hydrogels and elastomers is usually a synergistic action of two or more adhesion mechanisms.

### 2.1. Non-Covalent Bonding

**Figure 2:**
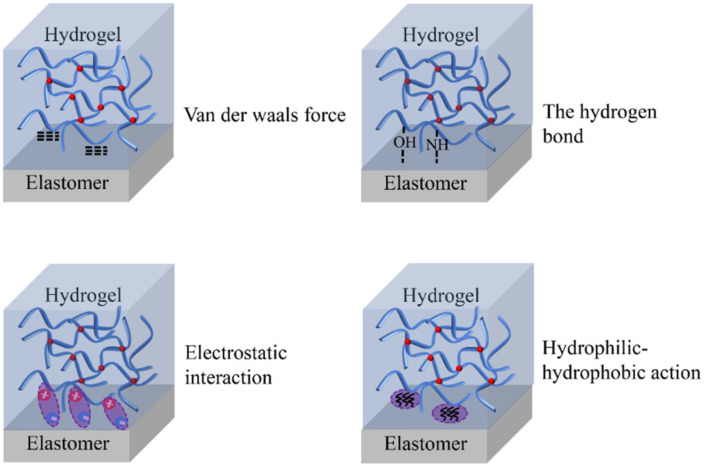
Hydrogel–elastomer adhesion mechanism based on physical interaction.

Non-covalent interactions, such as electrostatic interactions, Van der Waals interactions, hydrogen bonding, and hydrophilic–hydrophobic interactions, are generally used to establish adhesion between hydrogels and elastomers (Figure 2). Compared with covalent bonds, this non-covalent interaction is so weak that it is reversible [45]. Hence, a reversible hydrogel–elastomer interface adhesion structure is usually produced by the cooperative work of various non-covalent interactions [46]. These non-covalent interaction mechanisms have inspired the bioelectronic design of new biomimetic techniques, making adhesion reversible and robust at the interface between hydrogels and elastomers [47,48,49]. Hydrogen bonding involves an H atom in the hydrogel–elastomer polymer chain, which bonds to a relatively electronegative atom and another atom containing a lone electron pair. In general, single hydrogen bonds are weak, with bonding strength ranging from 1 to 10 kt [50].

Additionally, the formation of hydrophilic and hydrophobic interaction originates from the water-agglomeration tendency of the hydrophobic molecules. In the case of chemical linkage between hydrophobic molecules and hydrophilic hydrogel chains, there is an aggregation tendency of hydrophobic polymer chains (two or more) at the hydrophobic sites, which plays a cross-linking role. A hydrophobic group is usually an alkyl chain comprising C atoms. Although hydrophobic interactions have been utilized to produce self-healing tough hydrogels, their application for powerful hydrogel adhesion has never been documented.

### 2.2. Covalent Bonding

**Figure 3:**
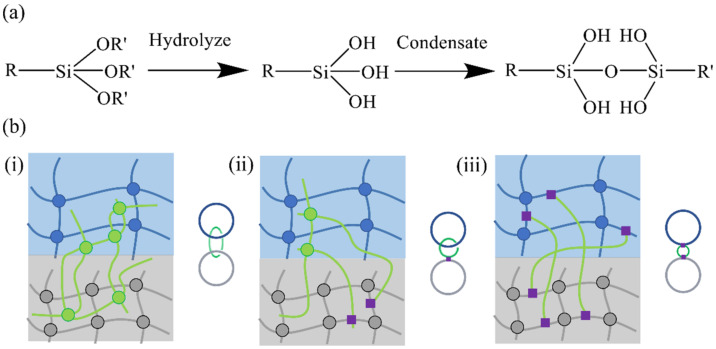
Two hydrogel–elastomer adhesion mechanisms: (**a**) Covalent bonding (reproduced with permission [55] Copyright 2018, Spring Nature). (**b**) Topology adhesion. (i) A third network is in topological entanglement with the two pre-existing networks; (ii) a third network is in topological entanglement with the hydrogel network and strongly and sparsely bonded to the elastomer network; (iii) a third network is strongly and sparsely bonded to hydrogel and elastomer networks (reproduced with permission [56] Copyright 2018, Spring Nature).

Normally, hydrogels either contain abundant functional groups or are functionalizable to further react with substrates (elastomers), leading to covalent bonding at the interface to attain excellent adhesion. Many functional groups in hydrogels, such as hydroxyl, ether, amino, carboxyl, or catechol groups, can react with those at elastomer surfaces to form amide, imine, or other covalent bonds. The high energies of covalent bonds often ensure the intensity and stability of the interface. Common hydrogel–elastomer covalent bonds include carbon–carbon [38,51,52], amide [53,54], siloxane [55], and carbon–nitrogen, which are irreversible. The preceding covalent bonds cannot be reformed upon breakage during adhesion isolation. Among them, silane chemical bonds are the most mature, and many commercially available compounds have been derived from them. In the chemical reactions of the silane covalent bonds, the silicon atom is linked to three hydrolyzable groups (hydroxyl, acetoxy, and chlorine) and an organic functional group. During polymer network formation, the organic functional group covalently incorporates the trialkoxysilane into the network. The alkoxy group hydrolyzes to a silanol group in the presence of water. Later, the silanol group condenses to form the siloxane bond (Figure 3a). [55]. Because there are no precise requirements for organic functional groups, this strategy can be used for a wide variety of networks.

### 2.3. Topology Adhesion

The topological adhesion mechanism is different from the covalent bond mechanism, which is the adhesion between the hydrogel polymer and the surface of the elastomer, and strong adhesion is achieved through special chemical covalent bonds. The topological adhesion mechanism exists in the adhesion between three molecular networks, namely the hydrogel monomer network, the elastomer network, and the adhesive network.

In recent years, topological adhesion has been achieved by the diffusion of monomers into adhesives and in situ polymerization to form topological entanglement. Generally, there are three different types of topological networks formed at the interface between hydrogels and elastomers (Figure 3b) [56]: topological intertwining with two preformed hydrogel and elastomer networks (Figure 3b(i)) [57]; topological intertwining with one preformed hydrogel network and powerful bonding to another preformed elastomer network (Figure 3b(ii)) [39]; or powerful bonding to two preformed networks (Figure 3b(iii)) [58]. If the third network is topologically entangled with two of the preexisting networks, then the functional groups of the two preexisting networks are not required to achieve stretchability and strong adhesion [59]. Under these circumstances, a molecular architecture is also constituted by the third network. Powerful hydrogel–elastomer adhesion can also be achieved through topological adhesion.

## 3. Methods for Hydrogel–Elastomer Adherence

In recent years, revolutionary advances have been achieved in strongly adhering hydrogels to elastomers, where the synergy among multiple adhesion mechanisms is critical. Among them, the commonly used experimental methods corresponding to each mechanism are different, including the popular catechol chemical method [60,61,62,63,64,65], surface modification [31,52], and the topological entanglement method [66,67,68,69]. In this section, these frequently used experimental approaches are proposed to attain robust hydrogel–elastomer adhesion.

### 3.1. Plasma or Ozone Treatment Method

**Figure 4:**
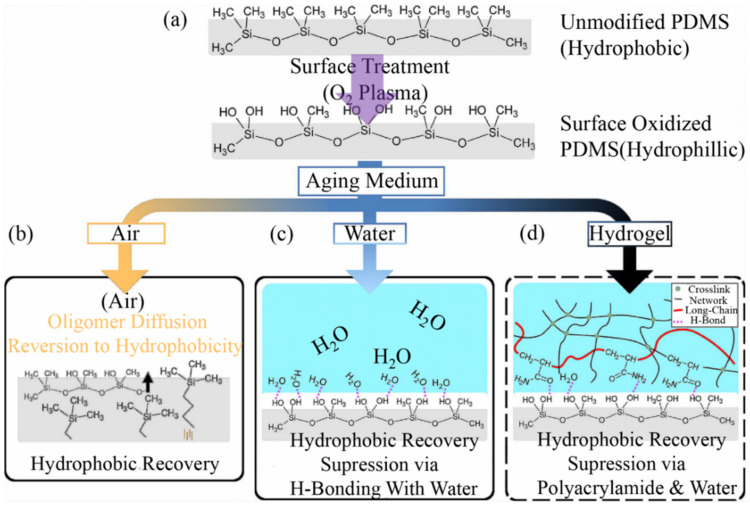
Plasma or ozone treatment method: (**a**) Treating the elastomer surface with oxygen plasma or ozone. (**b**–**d**) Illustration of the potential fate of a surface-oxidized PDMS sample, where the surface silanol groups are ether. Reproduced with permission [27] Copyright 2018, American Chemical Society.

Ozone or oxygen plasma can be used for elastomer surface processing, allowing adhesion between hydrogels and elastomers. The primary interaction is the Van der Waals force between the CH_3_ group of the polymer chain (hydrophobic) and water molecules. Hydrophilic treatment enables CH_3_-to-OH conversion, forming a hydrophilic layer (nanometric scale) and promoting the diffusion of hydrogel precursors [70,71]. Hydrophilic surfaces degrade easily in the air but remain in contact with water. However, such treatment often does not increase the adhesion energy. Suo et al. found that the power of hydrogel–elastomer adhesion was 15 J m^−2^ after six days of placement using the plasma treatment of the PDMS surface combined with the effect of the addition of non-ionic fluidized agent in hydrogels (Figure 4a) [27]. If exposed to the atmosphere, plasma-oxidized PDMS reverts back to a hydrophobic state in a matter of hours (Figure 4b). As the suppression of hydrophobic recovery has been observed with PDMS in contact with an aqueous medium (Figure 4c), the authors thought that the water in the hydrogel suppresses hydrophobic recovery through hydrogen-bond formation with the silanol groups at the PDMS surface (Figure 4d). Consequently, it is plausible that such a surface would also bind with other polar groups and enhance adhesion with other materials.

### 3.2. Catechol Chemical Method

**Figure 5:**
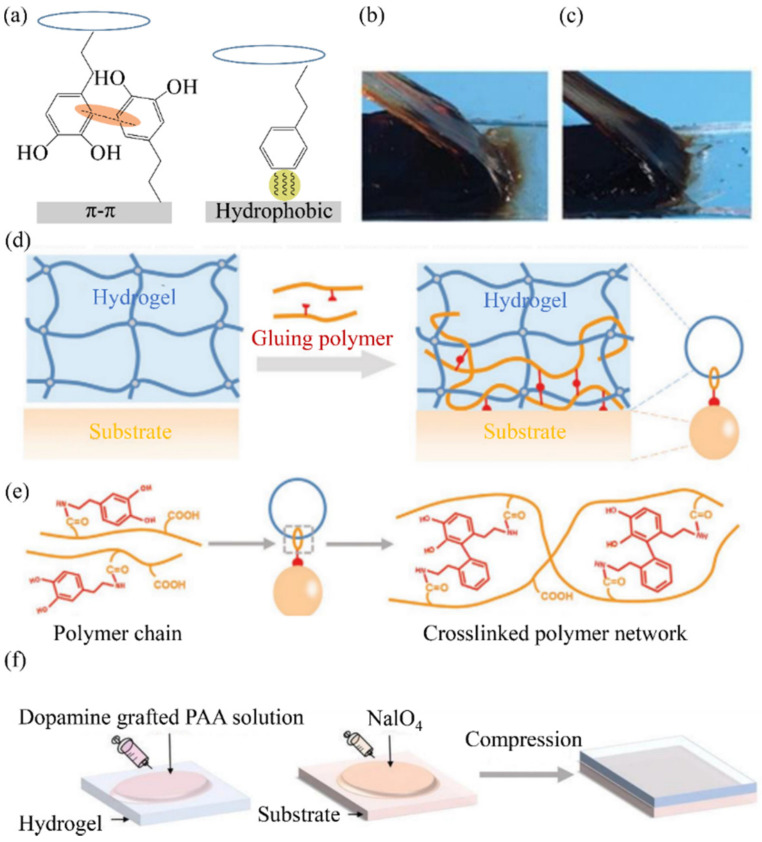
Catechol chemical method: (**a**) schematic representation of the two main catechol–surface interactions; (**b**,**c**) experimental photos; (**d**) schematic of the stitch-bonding mechanism; (**e**) the principle is shown using dopamine-grafted PAA as the NaIO_4_ and glue polymer as the trigger for intermolecular crosslinking; (**f**) universal “glue” based on catechol-modified polymer solution. Reproduced with permission [72] Copyright 2020, John Wiley and Sons.

In recent years, the characteristics of catechol, such as oxidative self-polymerization and multipurpose reaction, have attracted extensive attention from researchers, and research on mussel-induced adhesion has been widely reported. Through π–π accumulation or hydrophobic mechanisms, catechol and organic solids (involving alkane chains or benzene rings, such as plastics and elastomers) with the catechol benzene ring can be achieved (Figure 5a). Figure 5b,c shows that the PAAm (Polyacrylamide) hydrogels exhibit tough bonding interfaces with the tested adherends during a peeling process. The obvious large deformation in hydrogels and the brushed hair pattern appearing at the bonding front are direct indications of high interfacial toughness.

Additionally, metal-ion coordination bonds can be formed with metal substrates and form covalent bonds with skin tissues with active amines or sulfhydryl groups. Lu et al. developed a glue polymer by grafting catechol-containing minor molecules (e.g., dopamine) onto polymer chains pendant with carboxyl groups (e.g., polyacrylic acid, PAA) through EDC/NHS coupling agents (Figure 5d,e), then added NaIO_4_ as an oxidant to the adherend’s surface and pressed the hydrogel immediately with the glue polymer on the top of the adherend (Figure 5f), which achieved strong adhesion between the hydrogel and the elastomer, with possible bonding reaching ~300 J m^−2^ [72].

Catechol chemistry, which is derived from mussels, provides a valuable tool for to realize stitch-bonding adhesion. Catechol-modified polymers are widely used in the fabrication of tissue adhesive, [73] substrate coating [74,75], and self-adhesive hydrogels [76].

### 3.3. Surface Modification

**Figure 6:**
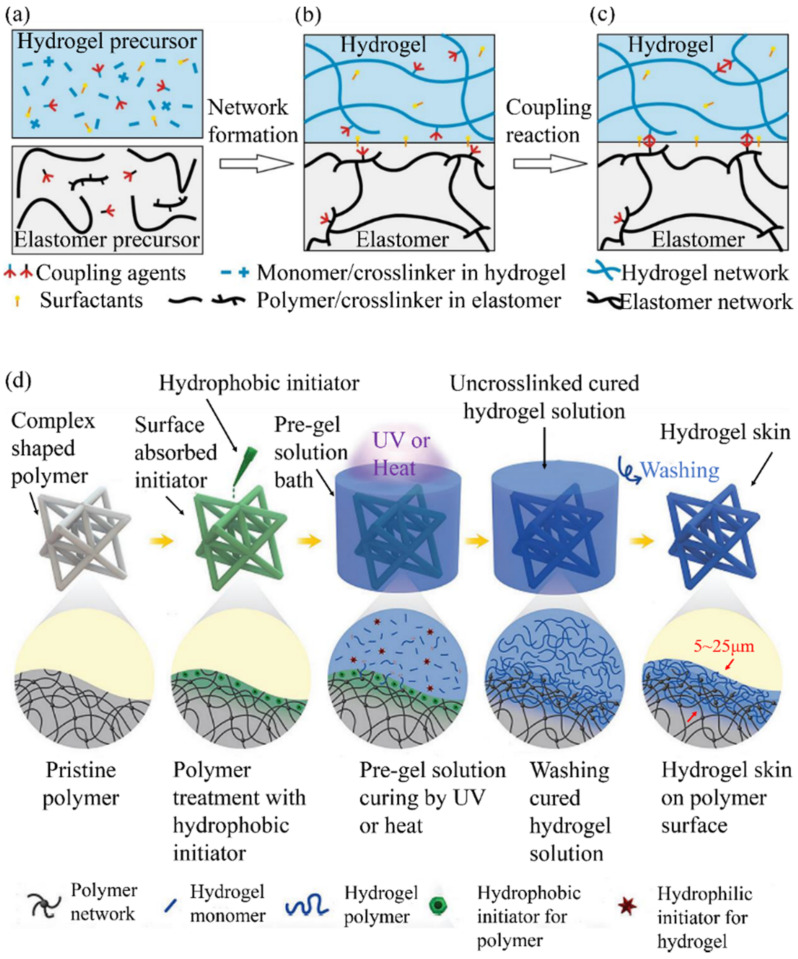
Surface modification: (**a**) silane coupling agent was added to the hydrogel–elastomer precursor; (**b**) by adjusting the dynamics, the coupling agent copolymerized; (**c**) after condensation, cross linking was generated between the hydrogel and the elastomer (reproduced with permission [55] Copyright 2018, Spring Nature); (**d**) hydrophobic benzophenone photoinitiator is adsorbed onto the target substrate’s surface through diffusion or an extra primer. The subsequent curing of a hydrogel precursor on the treated substrate enables a hydrogel coating to be strongly bonded to the substrate. Reproduced with permission [77] Copyright 2018, John Wiley and Sons.

The most common surface-modification strategy is grafting a hydrophilic polymer chain (e.g., poly (N-vinylpyrrolidone) or poly (ethylene oxide)/poly (ethylene glycol)) onto the polymer surface. The resulting elastomer surface is hydrophilic and colored due to the absorbent action of the grafted polymer, showing better antifouling properties than the uncoated surface. Suo et al. reported a method in which a silane coupling agent was added to the precursors of hydrogels and elastomers (Figure 6a). The coupling agent copolymerized (Figure 6b) by changing the kinetics and, after condensation, cross-linked the hydrogel–elastomer (Figure 6c) [55].

Zhao et al. [77] reported an uncomplicated yet effective strategy for interpenetrating crosslinked hydrophilic polymers (namely “hydrogel skins”) into the surfaces of various polymers, including silicone rubbers, polyurethane, PVC, nitrile rubber, and raw rubber with arbitrary states. Due to the unique combination of hydrophobic (i.e., water-insoluble) initiators that are absorbed into the polymer surfaces and hydrophilic (i.e., water-soluble) initiators that are dissolved in hydrogel pre-gel solution, the hydrogel skins can be formed in situ on the surfaces, conformally adjusting to the complex and fine geometries of the polymer substrates. They exhibit micrometer-scale tunable thickness that ranges from 5 to 25 µm with tissue-like softness (Young’s modulus ≈ 30 kPa), and the mechanical robustness is determined by the resultant hydrogel.

### 3.4. Topological Connection Method

**Figure 7:**
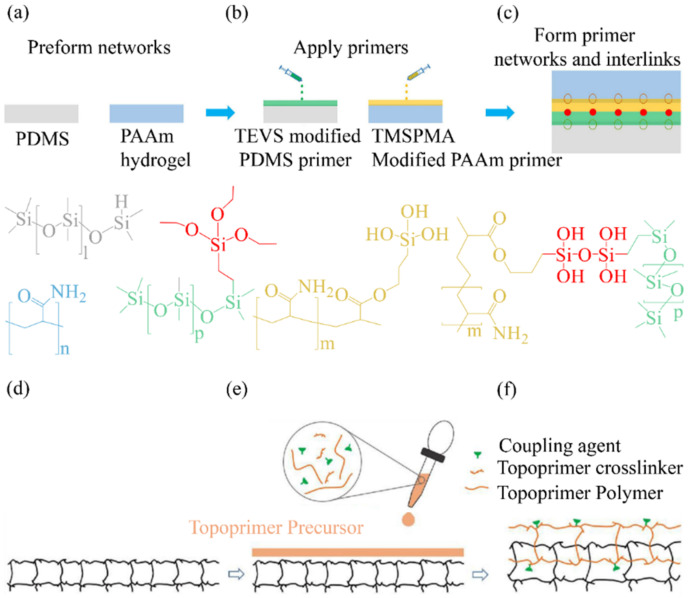
Topological connection method: (**a**–**c**) dual-primer adhesion between preformed PDMS elastomer and preformed PAAm hydrogel; reproduced with permission [78] Copyright 2020, Elsevier Ltd.; (**d**–**f**) the functional groups are sewn onto a substrate of an entropy polymer network. A top primer precursor contains a polymer, a cross-linking agent, and a coupling agent. Reproduced with permission [79] Copyright 2020, Springer Nature.

As the chemical adhesion between hydrogels and elastomers depends on special functional groups, how can we powerfully adhere two hydrogels to elastomers with no special functional-group binding? A “double-primed” approach to topological adhesion was described by Cheng et al. [78] in which the PAAm hydrogel and PDMS elastomer are prefabricated without functional groups for adhesion (Figure 7a). First, the TEVS (Triethoxyvinylsilane)-modified PDMS (Polydimethylsiloxane) primer was deposited onto the preprepared PDMS surface, and the TMSPMA-modified PAAm primer was deposited onto the preprepared PAAm surface (Figure 7b). After curing, a network of TEVS-modified PDMS primers was established, which exhibited topological intertwining with the preprepared PDMS network. Meanwhile, another network of TMSPMA (3-(trimethoxysilyl)propyl methacrylate)-modified PAAm primers was established, demonstrating topological intertwining with the preprepared PAAm network (Figure 7c). The silanes on the TEVS and TMSPMA groups hydrolyze to the silanol groups and condense with each other to form a covalent chain (namely the siloxane bond) between the two primer networks. This results in a strong and stretchable adhesive force up to approximately 140 J m^−2^.

Yang et al. [79] developed a universal approach for implementing topological connections, which they called “Topological prime” (Figure 7d–f). The functional groups are sutured onto a substrate of an entropic polymer network. A top primer precursor comprises a polymer, a cross-linking agent, and a coupling agent. When the precursor is applied to the elastomer substrate, after curing, the cross-linking agent connects the topological primer polymer into a network, which forms topological entanglements with the elastomer substrate network. In contrast, the coupling agent connects the topological primer network to the functional group. The effectiveness of the topological primer was proved by filling the hydrophilic coating with arbitrarily shaped hydrophobic elastomers.

## 4. Applications in Bioelectronics

Hydrogel–elastomer hybrid materials have been used extensively in the field of bioelectronics to simulate neuromuscular, artificial skin, and neurosensory systems, etc. The ion signals in hydrogels play an important role in neural perception. The ionizing device simulates the function of the skin, axon, and muscles due to the hydrogel–elastomer hybrid material but cannot simulate its anatomical structure [80]. Additionally, the signal can only be transmitted through ions and electrons. In this section, three common applications of hydrogel–elastomer mixtures in the field of bioelectronics are described.

### 4.1. Artificial Muscles 

Artificial muscle is a general term for a class of materials and devices imitating the way that living organisms’ muscles move. After an artificial muscle is subjected to outside stimuli (such as voltage, current, strain, temperature, or light), it reversibly shrinks, expands, rotates or merges the three elemental actuation reactions (contraction, expansion, and rotation) to achieve other kinds of movements. Artificial muscle actuators can be divided into mechanical, material, and biological types according their matrix composition. Mechanical artificial muscle actuators mainly include pneumatic artificial muscles (PAMs), electroconstrictors, and magnetoconstrictors. Material-type artificial muscle-actuator materials are mainly represented by shape memory alloys (SMAs), electrostrictive polymers (EAPs) [81,82], piezoelectric ceramics (PZT), magnetostrictive polymers, functional gels, liquid crystal elastomers, etc. A common feature of such artificial muscle actuators is that by simulating the working characteristics of animal muscle contraction to generate driving force, the material’s internal components undergo physical changes under different external controls (such as voltage, current, and pH), which generate driving force and deformation. Biological artificial muscle actuators are in the lab development stage, primarily using animal cells as actuators.

**Figure 8:**
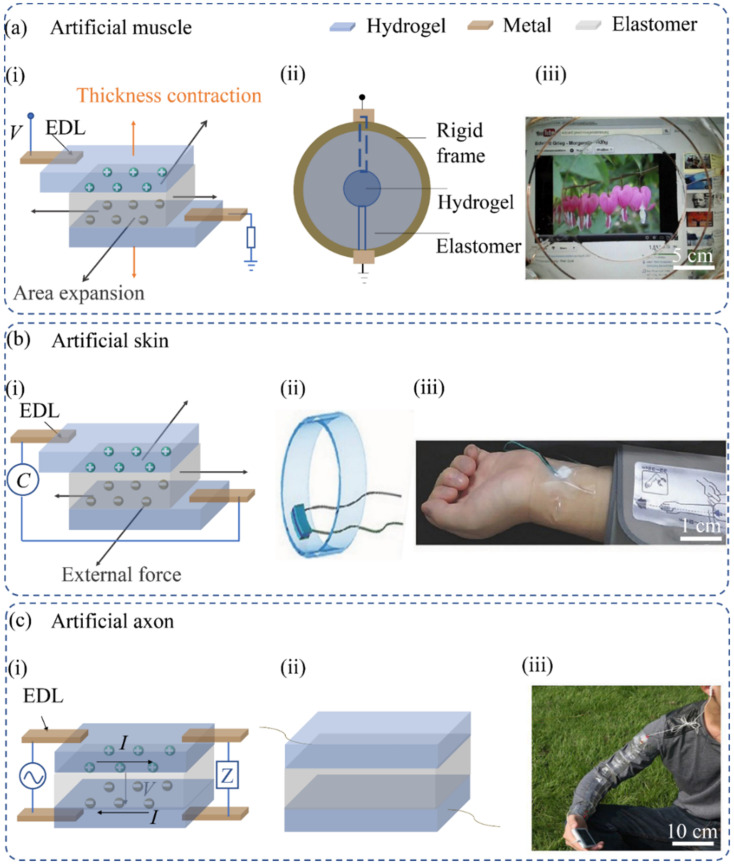
Applications in bioelectronics: (**a**) Artificial muscle mechanism. (i) Artificial muscle composed of two layers of ionized hydrogel and VHB. (ii) To realize an artificial muscle, a sheet of elastomer is radially prestretched and fixed to a circular rigid frame. (iii) Photos of the artificial muscle. Reproduced using permission [83] Copyright 2013, The American Association for the Advancement of Science. (**b**) Artificial skin mechanism. (i) Artificial skin composed of two hydrogels and an elastomer. (ii) The structure of pressure sensors. (iii) Photos of pressure sensors (ionic skin); reproduced using permission [13] Copyright 2014, John Wiley and Sons. (**c**) Artificial axon mechanism. (i) Artificial axon composed of two layers of hydrogel and elastomer. (ii) Structure of ionic cable. (iii) Photos of ionic cable (artificial axon); reproduced using permission [88] Copyright 2015, Elsevier Ltd.

The preparation of actuators is discussed in this paper from the perspective of dielectric elastomers that are composed of hydrogel–elastomer hybrid materials in electroconstrictive polymers (EAPs). The artificial muscle consists of an elastomer sandwiched between two hydrogels and linked to a power supply via a metal wire (Figure 8a). The metal and the hydrogel interface form an electrical double layer (EDL) [83]. The hydrogel generates ions when a voltage is adopted between two metal wires. Movable ions of opposite polarity gather at the interface between the hydrogel and the elastomer, leading to a reduction in thickness and an increase in the area of the elastomer. An elastomer is prestretched radially and fixed to a circular rigid frame to obtain the artificial muscle. In addition, a thin layer of hydrogel is attached to both sides of the elastomer at the center. Pelrine and colleagues formed the first artificial muscle to convert electrical voltage into mechanical motion [84]. Suo et al. [57] developed an artificial muscle assembled from two layers of a mixture of ionized hydrogel and VHB. 

Actuators made from hydrogel–elastomer hybrid materials provide mechanical motions commonly driven by the relatively large hydrogel–elastomer adhesion [85]. The strong hydrogel–elastic adhesion force satisfies the deformation requirements of the actuator in different application scenarios (photochromic, electrochromic, smart window, and display) [86,87]. The displays and hydrogel smart windows are soft and stretchable, allowing seamless interfaces for potential human–machine interactions [1]. The above artificial muscles, known as dielectric elastomer actuators, have witnessed considerable development and enable an extensive range of applications, including soft robotics, prosthetic devices, and adaptive optics.

### 4.2. Artificial Skin

Human skin is a retractable, large-area sensor of pressure, deformation, temperature, and humidity. The above properties have stimulated artificial skin development, allowing wearable or implantable electronic products to be used for entertainment and healthcare by employing stretchable electronic conductors or stretchable ion conductors, such as hydrogels. Conventional artificial skin uses electrons as charge carriers. When an external force is applied to capacitive or resistive bionic skin, the external force deforms the material, resulting in a change in resistance or capacitance. Common sensing materials using electrons as carriers include carbon nanotubes, graphene, gold, silicon, and platinum. For instance, Zhang et al. [89] fabricated biomimetic skin for mechanosensing with a highly conductive membrane and PDMS. The skin mainly uses graphene and carbon nanotubes as sensing materials and uses microstructured PDMS films as flexible substrates. These materials are suitable for electronic signal transmission; they meet the basic requirements of stretchability and electrical conductivity, but it is difficult to meet other requirements in specific applications, such as the biocompatibility required for biological signal monitoring [90], transparency [91], low elastic modulus, and mechanical adaptability.

To meet these challenges, it is crucial to develop new materials for bionic skin. Suo et al. were the first to develop an ion-electronic artificial skin consisting of an elastomer sandwiched between two hydrogels [92]. The two hydrogels are associated with the capacitive meter by two metal wires. The artificial skin is mostly covered in two extra layers of elastomer for electrical insulation and water retention (Figure 8b). Both elastomers and hydrogels are transparent and stretchable, and the wires are outside the active area, enabling the ion-electronic artificial skin to become transparent and stretchable [13,93]. The contact between the hydrogel and the wire is in-series with the elastomer capacitor to form two EDL capacitors. The equivalent capacitance is determined by the εeAe/He of the elastomer, which is the elastomer’s dielectric constant, the elastomer’s area, and the elastomer’s thickness. If the elastomer is stretched or pressed, its thickness decreases, and the area increases. The change in shape leads to a change in capacitance, which is recorded by the capacitometer. Unlike artificial muscle, which is deformed by high pressure, artificial skin is also deformed by applied force. The corresponding capacitance change can be calculated at a voltage of 1 V. Hydrogel ion-electronic artificial skin can sense single-touch events, sense multiple touches, and self-heal in the deformed state [17]. Hydrogel–elastomer hybrid-material-based artificial skin is also designed to sense changes in electrical resistance. Although current hydrogel ion-electronic artificial skin can only feel pressure and deformation, hydrogels can also be applied to convert changes in temperature and humidity into electrical signals that can mimic the role of human skin.

An ubiquitous usage has been found for hydrogel adhesion in recording electrodes and commercially available epidermal stimulation. Skin–electrode interfacial impedance is significantly affected by various factors, including conformal contact with the skin and the degree of epidermal hydration [94]. Hydrogel–elastomer adhesion is particularly suitability to ensure hydrated epidermis and conformal contact and has therefore been widely adopted in EEG’s various forms, EMG, ECG, and TENS electrodes. More recent advances in epidermal bioelectronic devices also benefit from hydrogel adhesion as a unique bridging interface to skin, including long-term conformal EMG sensors [95] and highly stretchable wearable devices.

### 4.3. Artificial Axon

Axons carry ion signals and coordinate perception, decision making, and function. The artificial axon mimics its process and some of its anatomical aspects. Artificial axon materials have ranged from the initial silica gel to the current tissue-engineering materials with varying functions. These materials can be roughly divided into metal materials, degradable natural macromolecular materials, non-degradable materials, and synthetic materials. The electrospinning method [96] is the main axon preparation method reported in the literature, as well as the weaving method [97], the pore-forming method, agent leaching, freeze drying, hydrogel injection [98], and rapid prototyping [99]. In the following section, artificial axons fabricated from hydrogel–elastomer materials will be introduced.

In an ionic electron artificial axon, two layers of hydrogel are divided by a layer of elastomer (Figure 8c), which is driven by the myelinated axon structure [88], with a saline solution as the electrolyte and a myelin sheath (the fatty sheath of the axon) as the dielectric shell. The electrolyte and dielectric shell provide a fast path for the electrical signal. Yang et al. [57] prepared an artificial ion axon made from a mixture of hydrogel and VHB, which is highly transparent and has a better signal transmission rate of up to 100 MHz over 10 cm. In artificial axons, dielectric elastomers mimic myelin sheaths, and electrolytic hydrogels mimic body fluids. One side of the artificial axon is an input port and is connected to an external power supply, whereas the other side serves as an output port and is linked to load Z [100]. If the signal is changed, the artificial axon can transmit the signal from the input port to the output port. In the artificial axon (ionic cable) [101,102,103,104], two hydrogels are insulated by an elastomer layer. The input port is associated with the time-varying voltage source, V. The output port is connected to the impedance load, Z. In a myelin axon, the myelin sheath is dielectric, and the fluid is the electrolyte ionic current. The contact between the hydrogel and the wire forms four EDLs in the artificial axon. In more invasive applications, neural implant biocompatibility can be improved by hydrogel adhesion with matching mechanical properties by attenuating neuroinflammatory responses. Soft poly (ethylene glycol) [94] and stronger poly (vinyl alcohol) [95] hydrogel adhesion implants (e.g., silicon and PDMS) decrease the extent of glial scarring and nervous cell loss by effectively minimizing the induced strain field throughout brain micromotions.

## 5. Conclusions and Outlook

In the present work, we reviewed the recent research progress in chemical hydrogel–elastomer adhesion, including the hydrogel–elastomer adhesion mechanism, preparation strategy, and applications. Hydrogels have excellent conductivity and transparency and high elasticity, water loss, freezing properties. Therefore, the combination of an elastomer and a hydrogel can fully retain their respective advantages and avoid the disadvantages of increased water loss and freezing of hydrogels. Hydrogels are hydrophilic, whereas elastomers are hydrophobic; the adhesion between them is weak, so determining how to improve the adhesion energy is a top priority. We need to research the adhesion mechanism and method between the two to solve this problem. Presently, the commonly used adhesion mechanisms include non-covalent mechanisms, covalent bonding, and topological adhesion. However, each of these mechanisms has some limitations; for example, determining how to combine the advantages of various adhesion mechanisms to improve the hydrogel–elastomer adhesive energy warrants in-depth study by many scholars. This also poses a challenge to the present adhesion mechanism between hydrogels and elastomers. Additionally, different methods are derived based on the adhesion mechanism, and these methods also have certain limitations, like the mechanism. Although many methods have been reported to improve the hydrogel–elastomer adhesion energy, a general strategy with a simple operation is lacking. The emergence of such a strategy is bound to greatly reduce the difficulty of hydrogel–elastomer adhesion.

Although some progress has been made in hydrogel–elastomer adhesion, further research in this field is urgently needed to promote its practical application. In addition to the challenges of adhesion mechanisms and the methods mentioned above, hydrogel–elastomer hybrid materials have unique tasks to solve in the bioelectronics field [105,106,107]. First, the stability of hydrogel–elastomer hybrid material is important for bioelectronics [108]. For instance, freezing and water loss are issues that must be addressed. When hydrogels lose water or freeze at low temperatures, they become hard and brittle without flexibility and biocompatibility or cannot maintain electrical conductivity. Some recent studies have used several methods, such as hydrogel modification or the substitution of solvents, to reduce the water loss rate and achieve anti-freezing. However, these methods can affect the electrical conductivity and mechanical properties of materials [109,110]. Secondly, the toughness and low strength of hydrogels need to be improved. There are many ways to improve the toughness and strength of hydrogels, such as double-network hydrogels and nanocomposite hydrogels. Beyond that, much of the work in hydrogel bioelectronics has focused on material development, with only proof-of-concept-level demonstrations. However, successful implementation ultimately depends on application-technology device-level transformation. Not surprisingly, the upcoming implementation of device-level hydrogel bioelectronics is likely to create more new issues, including higher mechanical and electrical performance requirements and integrated device assembly and manufacturing.

Of course, these challenges also provide future development with adequate development space; further improve electrical and hydrogel mechanical performance, which is likely to be a clear development direction; and add new features, such as biodegradability, to create new opportunities. The interface assembly between hydrogels and diverse instruments and tissues is an open question in this area. Here, we classify hydrogel bioelectronics future directions into three broad categories: (i) the growth of hydrogels with upgraded performance, (ii) integration between tough hydrogels and other device components, and (iii) advanced manufacturing approaches for hydrogel bioelectronics instruments. In future development, formidable challenges and tasks will be faced in hydrogel bioelectronics, but at the same time, it will also enable the seamless integration of electronics and biology with exciting prospects and provide new directions for the sustainable development of bioelectronic devices.

## Data Availability

The data used to support the findings of this study are included within the article.
